# The Impact of Wartime on Perinatal Outcomes in Kharkiv

**DOI:** 10.7759/cureus.94327

**Published:** 2025-10-11

**Authors:** Igor Lakhno

**Affiliations:** 1 Obstetrics and Gynecology, Kharkiv National Medical University, Kharkiv, UKR

**Keywords:** autonomic nervous regulation, fetal growth restriction, fetal heart rate variability, internally displaced persons, wartime

## Abstract

Objective: The armed conflict changed the humanitarian situation in the frontline areas in Ukraine. Kharkiv is a city located close to the frontline since the beginning of the war. The influence of war on fetal health and perinatal pathologies is still not clear.

The study was focused on the association between maternal heart rate variability (HRV), fetal HRV, gestational age at birth, neonatal biometric parameters, and Apgar scores in pregnant women from Kharkiv and internally displaced persons (IDPs).

Methods: This study was performed among 39 patients in 22-30 weeks of gestation who were enrolled in the study. The 20 residents were included in Group I. The 19 pregnant IDPs were included in Group II. Fetal HRV variables were obtained from an RR interval derived from the maternal abdominal wall via noninvasive fetal electrocardiography. The study protocol also included the term at birth, neonatal weight, length, head circumference, and Apgar score.

Results: The term of birth was significantly higher in Group I. PB occurred in 10% of Kharkiv residents. PB rate was higher in IDPs 42.1% (p= 0.0310). Group I newborns showed superior biometry to those born to IDPs. A moderate positive correlation was observed between fetal AC (acceleration capacity) and Apgar score (r = 0.34, p = 0.034), as well as between fetal DC (deceleration capacity) and Apgar score (r = 0.37, p = 0.019). The logistic regression model with the neonatal body weight showed the relationship with the AC maternal and maternal status (resident or IDP). The fetal growth demonstrated coupling with maternal autonomic regulation and maternal status. Such relationships among IDPs were stronger.

Conclusions: The rate for PB was significantly higher in IDPs. The term of birth, parameters of neonatal body weight, and body length were higher in the residents’ group. Fetal AC/DC demonstrated a moderate positive correlation with Apgar score. Therefore, these variables could be of use for monitoring fetal health. Maternal AC impacted neonatal body weight. The mechanism of the relationship between maternal sympathetic regulation and fetal growth was not understood. Maternal IDP status had a relationship with neonatal body weight. The wartime conditions affected fetal growth and maturation negatively in IDPs.

## Introduction

The armed conflict changed the humanitarian situation in the frontline areas in Ukraine. Kharkiv is located close to the Russian border, and it is a hub for internally displaced persons (IDPs) from Eastern Ukraine. The female population suffers from a stressogenic environment. Maternal comorbidities and gestational pathologies in Kharkiv showed notable deviations [1]. An increase in endocrine disorders and urinary tract infections was observed. Preterm birth (PB) is a result of disturbed placental function, maternal diseases, or cervical incompetence. Prematurity is a cornerstone of modern perinatology. A short cervix measured via ultrasound is a prognostic marker for PB [2]. This sign is independent of the reason for PB and is thought to be universal. The prediction and prevention of PB is a challenge during the war.

The influence of war on fetal health is still not clear. However, fetal programming (Barker’s hypothesis) arose in the Netherlands during World War II [3]. It postulates that low weight at birth is a trigger for cardiovascular, metabolic, and neurological disorders in the future. The weak point of this theory is the absence of differentiation between low birth weight due to prematurity or fetal growth restriction. Fetal neurological maturation is a crucial event in the process of its growth and development. Heart rate variability (HRV) reflects the autonomic control of the cardiovascular system [4]. Fetal HRV is not a mirror of maternal autonomic function. Coupling of maternal and fetal hemodynamic fluctuations has been observed in healthy pregnancies. Fetal non-invasive electrocardiography (FNI-ECG) is an approach used to measure fetal electrophysiological cardiac signals [5]. 

The study was focused on the association between maternal heart rate variability (HRV), fetal HRV, gestational age at birth, neonatal biometric parameters, and Apgar scores in pregnant women from Kharkiv and internally displaced persons. 

## Materials and methods

Study design

This descriptive cross-sectional study was performed among pregnant women admitted to Kharkiv Municipal Perinatal Center between 1 September 2024 and 30 June 2025. The research was performed within the project of the Department of Obstetrics and Gynecology No. 3 of Kharkiv National Medical University (0123U104315), developing a system for predicting, preventing, and treating pregnancy, labor, and postpartum complications in women affected by wartime stress.

Patient recruitment

Patients from the department of maternal-fetal medicine were selected randomly. Inclusion criteria included pregnant women who were residents of Kharkiv or IDPs. Exclusion criteria included chromosomal abnormalities, multiple pregnancies, and preexisting severe medical disorders such as diabetes mellitus, metabolic syndrome, cardiac diseases, renal disease, and thyrotoxicosis. Ethics approval was received from the Research Council and Ethical Committee of Kharkiv National Medical University. Informed consent was obtained from all the patients. The eligible participants were informed about the study’s methodology, aims, objectives, indications, and potential complications before enrollment.

In total, 39 patients in 22-30 weeks of gestation were enrolled in the study. The 20 residents were included in Group I. The 19 pregnant IDPs were included in Group II. All patients included in the study have no personal history of spontaneous miscarriages or PTB.

Data collection

The data was obtained from the hospital automation system. The technique of automated numbers was used. The maternal and fetal HRV variables were obtained from an RR-interval time series derived from the maternal abdominal wall via FNI-ECG. The equipment “Cardiolab Baby Card” (Scientific Research Center “KhAI-Medica,” Ukraine) was used in the study. The registration was carried out over 30-60 minutes. The stress index (SI) was selected for evaluation among all linear maternal and fetal HRV variables. SI = AMo (%) / (2×Mo×Var); Var = NNmax - NNmin; AMo (the most frequent value of NN interval or the highest column in the histogram) - the number of NN intervals included in the pocket corresponding to the mode measured in percentages (%). The obtained fetal RR interval time series was transformed into a cardiotocographic (CTG) tracing. The following CTG parameters were determined: short-term variation (STV) and long-term variation (LTV). Maternal and fetal AC/DC (acceleration and deceleration capacity) variables were also measured [6]. 

The study protocol also included the term at birth, neonatal weight, length, head circumference, and Apgar score.

Statistical analysis

IBM Corp. Released 2022. IBM SPSS Statistics for Windows, Version 28. Armonk, NY: IBM Corp. was used for statistical analysis. Results were presented as means and standard deviations for numerical variables and frequencies and percentages for categorical data. The suitability of numerical variables to the normal distribution was evaluated using skewness values and histograms.

The independent sample t-test was used to compare numerical variables that fit a normal distribution. Variables exhibiting non-normal distribution were evaluated using the Mann-Whitney U test. Categorical variables were compared using chi-square or Fisher’s exact test. The 2x2 table p-value was calculated. Spearman or Pearson correlation was used, based on data distribution, to assess relationships between numerical variables. Logistic regression was used for multivariate analysis with the entered model method. Statistical significance was set at p < 0.05.

## Results

The mean age of the women was almost similar in Group I (25.6±5.8) and Group II (26.2±7.3) (p>0.05). The rate of primigravidae was 45% and 57.9% in Group I and Group II, respectively. The results obtained showed that maternal and fetal HRV variables did not differ significantly between the population of the study groups (Table 1). The term of birth was significantly higher in Group I. PB occurred in 10% of Kharkiv residents. PB rate was higher in IDPs 42.1% (p= 0.0310). The variables of neonatal body weight and body length differ significantly. Group I newborns showed superior biometry to those born to IDPs. However, the difference in head circumference and Apgar score between Group I and Group II did not reach statistical significance.

**Table 1:** The variables of maternal and fetal HRV, term of birth, neonatal biometry parameters, and Apgar score. The mean values of maternal and fetal AC/DC, SI, fetal STV and LTV, term of birth, and neonatal biometry. The evident changes in terms of birth, neonatal body weight, and body length (p<0.05) between Kharkovites and IDPs. AC: Acceleration Capacity, DC: Deceleration Capacity, SI: Stress Index, STV: Short-Term Variation, LTV: Long-Term Variation

Variable, units of measure	Status	Frequency	Mean	Std. deviation	Minimum	Maximum
AC maternal, ms	Resident	20	8.25	2.42	4.25	12.82
	IDP	19	8.54	2.85	4.44	14.82
	p=0.7334					
DC maternal, ms	Resident	20	8.17	2.05	4.94	11.49
	IDP	19	8.9	3.34	4.53	17.13
	p=0.4133					
AC fetal, ms	Resident	20	1.84	0.5	0.81	3.07
	IDP	19	1.85	0.67	0.77	3.71
	p=0.9580					
DC fetal, ms	Resident	20	2.18	0.7	0.78	3.61
	IDP	19	2.2	0.81	0.82	4
	p=0.9346					
SI maternal, ms	Resident	20	186.75	93.62	48	430
	IDP	19	162.79	98.4	54	457
	p=0.4408					
SI fetal, ms	Resident	20	1028.7	497.07	251	2167
	IDP	19	1061	713.27	349	3102
	p=0.8700					
STV, ms	Resident	20	6.65	2.44	1.7	12.3
	IDP	19	6.98	3.03	1.2	13
	p=0.7094					
LTV, ms	Resident	20	34.42	12.32	12.3	71
	IDP	19	36.04	13.68	9.3	58.7
	p=0.6995					
Term of birth, weeks	Resident	20	38.05	2.87	27	41
	IDP	19	34.84	3.37	26	39
	p=0.0028					
Body weight, g	Resident	20	3333.5	1011.48	440	5530
	IDP	19	2294.74	853.32	410	3500
	p=0.0014					
Body length, sm	Resident	20	52	7.22	29	62
	IDP	19	44.84	7.64	27	56
	p=0.0047					
Head circumference, sm	Resident	20	33.55	3.97	19	38
	IDP	19	31.32	4.19	19	36
	p=0.0963					
Apgar score, points	Resident	20	7.7	2.08	0	9
	IDP	19	6.74	2.05	0	9
	p=0.1553					

The analysis of linear correlation between the measured variables in the study population revealed observable patterns (Table 2). There was no detected correlation between maternal and fetal HRV. No association was found between fetal HRV and neonatal biometry variables. The term of birth demonstrated a strong positive correlation with biometry parameters and Apgar score. A moderate positive correlation was observed between fetal AC and Apgar score (r = 0.34, p = 0.034), as well as between fetal DC and Apgar score (r = 0.37, p = 0.019). 

**Table 2:** Linear correlation between maternal and fetal HRV, term of birth, neonatal biometry parameters, and Apgar score. The parameters of linear correlation showed a relationship between fetal biometric variables and Apgar score and between fetal AC/DC and Apgar score. AC: Acceleration Capacity, DC: Deceleration Capacity, SI: Stress Index, STV: Short-Term Variation, LTV: Long-Term Variation, HRV: Heart Rate Variability

Variables	Cor- relation	AC maternal	DC maternal	AC fetal	DC fetal	SI maternal	SI fetal	STV	LTV	Term of birth	Body weight	Body length	Head circum-ference	Apgar score
AC maternal	r	1	0.96	0.02	-0.01	-0.78	0.09	-0.17	-0.18	-0.02	-0.21	-0.15	-0.16	-0.08
	p		< .001>	.891	.953	< .001>	.572	.304	.276	.916	.203	.349	.326	.636
DC maternal	r	0.96	1	0.01	-0.02	-0.83	0.08	-0.16	-0.18	-0.04	-0.18	-0.13	-0.12	-0.07
	p	< .001>		.948	.888	< .001>	.641	.33	.287	.791	.286	.425	.462	.658
AC fetal	r	0.02	0.01	1	0.86	-0.16	-0.65	0.85	0.84	0.22	0.06	0.16	0.23	0.34
	p	.891	.948		< .001>	.329	< .001>	< .001>	< .001>	.183	.717	.317	.155	.034
DC fetal	r	-0.01	-0.02	0.86	1	-0.14	-0.63	0.9	0.84	0.2	0.05	0.15	0.21	0.37
	p	.953	.888	< .001>		.38	< .001>	< .001>	< .001>	.226	.746	.36	.208	.019
SI maternal	r	-0.78	-0.83	-0.16	-0.14	1	-0.07	0.01	0.07	0.04	0.04	0.07	-0.02	-0.03
	p	< .001>	< .001>	.329	.38		.658	.931	.673	.813	.787	.69	.904	.873
SI fetal	r	0.09	0.08	-0.65	-0.63	-0.07	1	-0.73	-0.8	-0.1	0.07	-0.09	-0.14	-0.24
	p	.572	.641	< .001>	< .001>	.658		< .001>	< .001>	.543	.675	.569	.382	.144
STV	r	-0.17	-0.16	0.85	0.9	0.01	-0.73	1	0.96	0.1	0.02	0.14	0.2	0.28
	p	.304	.33	< .001>	< .001>	.931	< .001>		< .001>	.53	.913	.389	.222	.09
LTV	r	-0.18	-0.18	0.84	0.84	0.07	-0.8	0.96	1	0.06	-0.03	0.1	0.16	0.23
	p	.276	.287	< .001>	< .001>	.673	< .001>	< .001>		.695	.857	.551	.319	.157
Term of birth	r	-0.02	-0.04	0.22	0.2	0.04	-0.1	0.1	0.06	1	0.85	0.83	0.68	0.73
	p	.916	.791	.183	.226	.813	.543	.53	.695		< .001>	< .001>	< .001>	< .001>
Body weight	r	-0.21	-0.18	0.06	0.05	0.04	0.07	0.02	-0.03	0.85	1	0.92	0.84	0.68
	p	.203	.286	.717	.746	.787	.675	.913	.857	< .001>		< .001>	< .001>	< .001>
Body length	r	-0.15	-0.13	0.16	0.15	0.07	-0.09	0.14	0.1	0.83	0.92	1	0.85	0.74
	p	.349	.425	.317	.36	.69	.569	.389	.551	< .001>	< .001>		< .001>	< .001>
Head circum-ference	r	-0.16	-0.12	0.23	0.21	-0.02	-0.14	0.2	0.16	0.68	0.84	0.85	1	0.72
	p	.326	.462	.155	.208	.904	.382	.222	.319	< .001>	< .001>	< .001>		< .001>
Apgar score		-0.08	-0.07	0.34	0.37	-0.03	-0.24	0.28	0.23	0.73	0.68	0.74	0.72	1
	p	.636	.658	.034	.019	.873	.144	.09	.157	< .001>	< .001>	< .001>	< .001>	

The logistic regression model with the neonatal body weight showed the relationship with AC maternal and maternal status (resident or IDP) (Table 3). The results indicated statistical significance for this model. The fetal growth demonstrated coupling with maternal autonomic regulation and maternal status. Such relationships among IDPs were stronger.

**Table 3:** Logistic regression model with neonatal body weight. The evident logistic regression was found between newborn body weight and maternal AC values and newborn body weight and maternal status (IDP). AC: Acceleration Capacity, DC: Deceleration Capacity, SI: Stress Index, STV: Short-Term Variation, LTV: Long-Term Variation, IDP: Internally Displaced Persons

Variable	Unstandardized Coefficients	Standardized Coefficients	Standard error	t	p	95% confidence interval for B	
Model	B	Beta				lower bound	upper bound
(Constant)	-2807.7		896.02	-3.13	.004	-4657.09	-958.31
AC maternal	-147.76	-0.36	67.32	-2.19	.038	-286.71	-8.81
DC maternal	95.56	0.25	64.07	1.49	.149	-36.67	227.79
AC fetal	-106.72	-0.06	225.66	-0.47	.641	-572.49	359.05
DC fetal	-1.43	0	214.94	-0.01	.995	-445.06	442.21
SI maternal	-1.21	-0.11	0.82	-1.47	.155	-2.9	0.49
SI fetal	0.23	0.13	0.14	1.63	.117	-0.06	0.52
STV	28.91	0.07	77.03	0.38	.711	-130.07	187.89
LTV	-7.75	-0.09	11.24	-0.69	.497	-30.96	15.45
Term of birth	36.34	0.12	41.78	0.87	.393	-49.89	122.57
Body length	95.72	0.74	20.05	4.77	< .001>	54.34	137.11
Head circumference	77.35	0.3	41.62	1.86	.075	-8.55	163.24
Apgar score	-106.78	-0.21	61.64	-1.73	.096	-234	20.44
IDP	-1516.72	-0.72	432.92	-3.5	.002	-2410.27	-623.17
Resident	-1290.98	-0.61	470.85	-2.74	.011	-2262.8	-319.15

## Discussion

The findings of the study emphasized the issues of fetal growth and PB in IDPs. Therefore, IDPs were identified as a target population for studying fetal programming and its potential effects on newborn health in later life [7]. Prematurity was a crucial issue for perinatal pathologies in IDPs. Residents had higher birth terms, neonatal weight, and body length, indicating that preventing PB contributes to better perinatal outcomes. The availability of vaginal progesterone, pessaries, or cerclage should be assessed in frontline cities. The nutritional issues and concerns in these areas should be evaluated.

The absence of coupling between maternal and fetal HRV may be attributable to the level of PB present in the study population. This finding supports the notion that the decoupling of maternal and fetal hemodynamic fluctuations was a component of the pathogenesis of PB. Disturbed placentation was a part of maternal and fetal autonomic malfunction in fetal growth restriction [8]. Following the identification of correlations between fetal AC/DC and Apgar scores, fetal AC/DC was found to be of use in the assessment of fetal distress. 

The found relationship between maternal AC showed the significance of maternal sympathetic regulation in fetal growth. Increased sympathetic branch activity has been reported to play a role in PB [9]. However, the mechanisms of these relations could not be explained. Therefore, maternal autonomic nervous regulation had multiple projections on perinatal outcomes.

The war changed the lives of pregnant women [10]. Some of them became refugees; some women changed their whereabouts within Ukraine and got IDP status. Wartime stress was a possible trigger for gestational complications and perinatal pathologies. The latest systematic review showed the involvement of wartime stress in altered neurodevelopment, mental disorders, and pathophysiological diseases [11]. The patterns of psychological disturbances based on negative perinatal experience were reported [12]. The opinion is that the spread of chemicals due to armed conflict in Ukraine caused a polluted environment and health issues among pregnant women and newborns [13]. One study showed the growth of maternal emergencies and maternal mortality due to limited access to medical services during the war in Ethiopia [14]. Mechanical traumas were found to be the main aspect of maternal complications in Gaza [15]. The issues of safe maternity and birth have not been adequately addressed yet. Probably, only peace could restore maternal health and beneficial perinatal outcomes in the future [16]. However, the negative trends in maternal health in Armenia, even after the war, persisted [17].

The impact of war was multiple. It negatively affected maternal and fetal health. The decline in neonatal birth weight during the war in Ukraine was supported [18]. The development of initiatives aimed at reducing pregnancy-related complications during periods of armed conflict represents a fundamental component of obstetric health care.

Limitations of the study

The restricted number of women included in the study was a limitation.

## Conclusions

The rate for PB was significantly higher in IDPs. The term of birth, parameters of neonatal body weight, and body length were higher in the residents’ group. Fetal AC/DC demonstrated a moderate positive correlation with Apgar score. Therefore, these variables could be of use for monitoring fetal health. Maternal AC impacted neonatal body weight. The mechanism of the relationship between maternal sympathetic regulation and fetal growth was not understood. Maternal IDP status had a relationship with neonatal body weight. The wartime conditions affected fetal growth and maturation negatively in IDPs. 
